# Correcting motion in multiplanar cardiac magnetic resonance images

**DOI:** 10.1186/s12938-016-0216-8

**Published:** 2016-08-08

**Authors:** Min Wan, Wei Huang, Jun-Mei Zhang, Xiaodan Zhao, John Carson Allen, Ru San Tan, Xiaofeng Wan, Liang Zhong

**Affiliations:** 1 Nanchang University, No. 999, Xuefu Dadao, Nanchang, People’s Republic of China; 2 National Heart Centre Singapore, 5 Hospital Drive, Singapore, 169609 Singapore; 3 Duke-NUS Medical School Singapore, 8 College Road, Singapore, 169857 Singapore

**Keywords:** Motion correction, Cardiac magnetic resonance (CMR)

## Abstract

**Background:**

Misalignment in cardiac magnetic resonance (CMR) images can adversely affect three-dimensional left ventricle modelling and downstream quantitative analysis. Currently, there are two types of approaches for dealing with realignment and motion distortion problems, one image based and the other geometry based. Image-based approaches are limited by the inherent non-homogeneity and anisotropy of CMR images. Geometry-based approaches rely on idealized models and over-simplified assumptions. This study was motivated by the need for a robust and effective approach for correcting motion related distortions due to misalignment in CMR images.

**Methods:**

A cine cardiac magnetic resonance image sequence was acquired using our routine clinical imaging protocol. The left ventricular endocardium was delineated manually with software assistance on all long and short-axis images. Long and short-axis contours were projected onto a patient-based coordinate system and then realigned using iterative registration. The realigned contour points were used to reconstruct the shape of the left ventricle for quantitative validation.

**Results:**

The method was tested on five myocardial infarction patients whose images showed substantial misalignment. Realignment time was about 16 seconds per case, using a 2.5 GHz CPU desktop with obvious elimination of the distortion in the reconstructed model. Using the long-axis contour as a reference in evaluating the reconstructed models, it was apparent that the models with realigned contours had better accuracy than the non-realigned ones.

**Conclusion:**

This study presents a novel, geometry-based method for correcting motion distortions in CMR images. The method incorporates (1) manual delineation, (2) registration based on a generalized, iterative closest point algorithm, and (3) reconstruction of the shape of the left ventricle for quantitative validation. The effectiveness of our approach is corroborated both visually and by quantitative assessment. We envision the use of our method in current clinical practice as a means of improving accuracy in the evaluation of cardiac function.

## Background

Cardiovascular disease (CVD) is the leading cause of death throughout the world and accounts for 17 % of deaths in the USA. Cardiac magnetic resonance (CMR) imaging has been an established method for evaluating cardiac function [1–3]. It is considered the gold standard in recent literature for evaluating left ventricular cardiac function [4, 5].

In current clinical practice, cine CMR images are usually acquired at multiple locations over multiple breathing cycles and typically include three orthogonal long-axis image sequences with parallel short-axis image sequences. All image sequence are acquired in separate breath-holds. Different diaphragm positions throughout the acquisitions and random patient motion results in image slice misalignment that undermines the accuracy of the CMR three-dimensional left ventricle (LV) model. Advances in image acquisition techniques give rise to the possibility of acquiring an entire cine CMR image in a single breath-hold. Temporal/spatial resolution would be reduced [6] but techniques could be implemented on other imaging modalities as well [7]. CMR imaging in a routine cardiac examination is unavoidably subject to slice misalignment due to the breath-hold and patient movement. Misalignment is a recognized problem among researchers [8, 9] with attempts at correction using manual realignment.

Attempts by researchers to address the misalignment and motion correction problems in the past decade have relied on a variety of approaches which can be categorized as essentially image based or geometry based. The image-based registration approach utilizes pixel similarity to register images, and has been studied extensively by the computer vision community. Ector et al. [8] registered consecutive short-axis slices to find an in-plane translation for each short-axis slice. Considering the relatively large spacing (typically 8 mm) between neighboring slices, the images to be registered could be considerably different, causing registration inaccuracies. Instead of directly registering short-axis images, Chandler et al. [10] registered each short-axis image to a three-dimensional isotropic volume image acquired specifically for the purpose of research. Similarly, Lötjönen et al. [11] acquired additional parallel long-axis images for registration which did indeed increase accuracy. However, the requirement for additional image acquisition in a routine clinical imaging protocol renders these approaches impractical in clinical practice. In Slomka [12] and Barajas [13] et al., short-axis images were registered with long-axis images by maximizing the similarity of pixels at the intersection of image planes. Elen et al. [14] presented a comprehensive method to register all images simultaneously while comparing performances from a variety of similarity based cost functions in earlier studies.

Geometry-based approaches initially extract geometric information regarding LV shape from the images and then utilize this information to realign the image slices. The initial geometry-based extraction step is usually a manual delineation of the LV contour on short- and long-axis images. Van Assen et al. [15] realigned the short-axis contours to have centroids coincide with the intersection of the long-axis imaging planes. This method is based on the assumption that the LV is nearly a symmetric shape, i.e., the combination of a cone and an ellipsoid. This idealized and oversimplified assumption was also used in [16]. Tan et al. [17] addressed the motion correction problem as the minimization of a certain energy function regarding curvature of the reconstructed LV shape. The assumption used is that the LV shape is convex for most vertices on the surface, which could be inaccurate or incorrect for highly variable cases—in particular, myocardial infarction patients with LV remodeling.

The image-based approaches could be made fully automatic and avoid the tedious LV segmentation task. However, motion correction based solely on images is intrinsically inaccurate due to the large slice spacing and the complex nature of images (heterogeneity, non-uniformity) as well as the existence of papillary muscles. Geometry-based approaches can be criticized regarding manual contour delineation-the geometric information extraction step.

We note that image realignment is only an intermediate task, and that obtaining the LV shape from the realigned images for the purpose of quantitative analysis [18–28] is the ultimate goal. In addition, contour delineation, the most tedious part of the image-based approaches, can be conducted at the second stage. Semi-automatic or automatic chamber segmentation has been intensively studied for decades and could significantly reduce processing time. This study was motivated by the need for a robust and effective approach for correcting motion related distortions due to misalignment in CMR images.

In this study, a novel method is proposed to correct breath-hold related or overall motion for multiplanar cine CMR images. Images were acquired using routine clinical imaging protocol. A stack of parallel short-axis images as well as three orthogonal long-axis images were acquired. LV endocardial contours were manually delineated on all images. After projecting the 2D in-plane contours into the 3D patient-based coordinate system, each short-axis contour was registered against the whole long-axis contours, followed by registering the long-axis contours to the whole short-axis contours. The above registration steps were iteratively repeated until convergence. The final registration configuration was applied to realign the contours. Both un-realigned and realigned contour points were utilized to reconstruct the LV shapes, respectively. The accuracy of reconstructed LV models were evaluated via comparing to the long-axis contours. A better coincidence of the reconstruction from realigned contours indicates the effectiveness of our method.

The remainder of this article is organized as follows. "Methods" section describes the methodology. "Results" section provides the experimental results and the validation. "Conclusions" section concludes this article.

## Methods

In this study, we tested the algorithm on five patients whose cine images had noticeable misalignment. The study was approved by the SingHealth Centralised Institutional Review Board for human research. All enrolled participants gave written informed consent. The MR data are deposited in hospital and available for research and education purposes. Cardiac related measurements for each patient are given in Table 1.

**Table 1:** Statistics on patients

Patient	Age	Height (cm)	Weight (kg)	RR (ms)	Heart rate (beats/min)	SBP (mmHg)	DBP (mmHg)	BSA $m^2$	Lvmass (g)
1	48	169	75	595	101	135	32	1.9	134
2	46	168	83.6	1220	49	122	78	2	116
3	63	159	59	900	67	119	71	1.6	104
4	56	166	88.2	650	92	140	88	2	162
5	72	165	77	690	87	136	77	1.9	113
Average	57	165	77	811	79	130	69	2	126

**Fig. 1:**
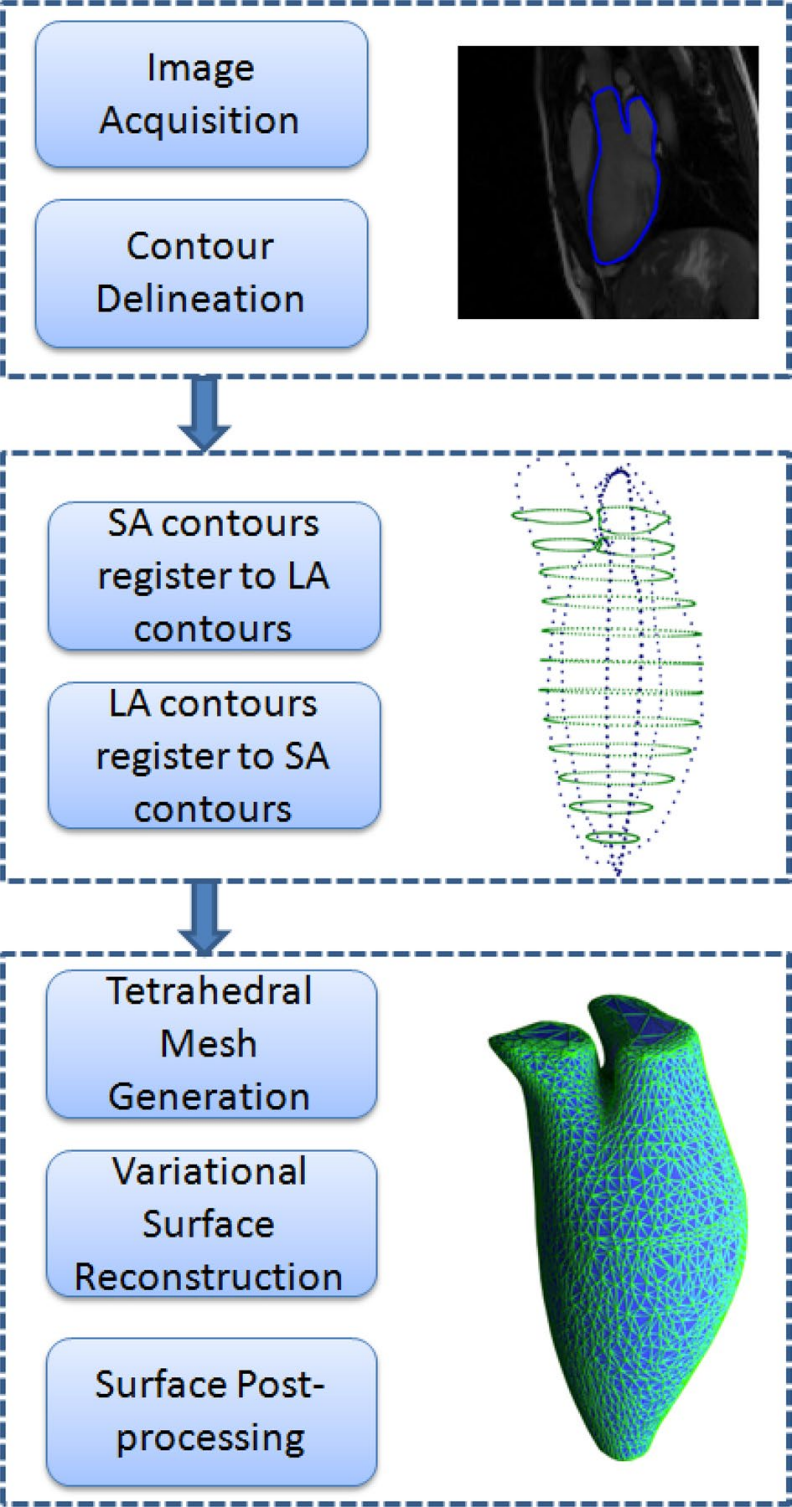
Flowchart of the present method. The flowchart illustrates the main three stages in the present method: (1) image acquisition and pre-processing; (2) iterative registration among contours; (3) LV shape reconstruction. *SA* short axis, *LA* long axis

The proposed method consists of three steps: (1) LV contours on the acquired multiplanar CMR images are segmented; (2) contours from different planes are registered in the 3D patient-based coordinate system using an iterative two-step approach; (3) the realigned contours are utilized to reconstruct the LV shape. The realignment task correcting for motion distortions is actually accomplished in the second stage—and this is where the typical literature description ends. The reason we add the last step is twofold: (1) reconstructing the LV shape is a natural subsequent processing step in quantitative analysis in cardiology; and (2) we can use the reconstructed LV shape to validate our method. The flowchart describing our method is shown in Fig. 1. Each step will now be described in detail.

### Image acquisition and contour delineation

All images were acquired on a 1.5T Siemens cardiac MR scanner using a routine clinical imaging protocol. Parallel short axis and long axis (two-chamber, three-chamber and four-chamber view) images were included. The number of short axis slices varied from 12 to 14 depending on the size of heart. Each image slice was acquired in a single breath-hold. The imaging parameters were as follows: field of view (FOV) = 320 mm, image size = 192 × 150, pixel spacing = 1.77 × 1.77 mm, slice thickness/spacing = 8/8 mm, TR/TE/flip angle = 68/1 ms/$70^{\circ }$. Short axis cine images had 22 phases while long axis images had 25 phases.

**Fig. 2:**
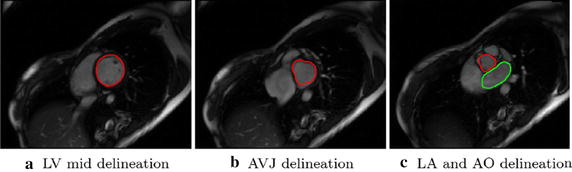
Short axis images and delineated contours. Short axis images at three slice locations were shown as well as the delineated contours

**Fig. 3:**
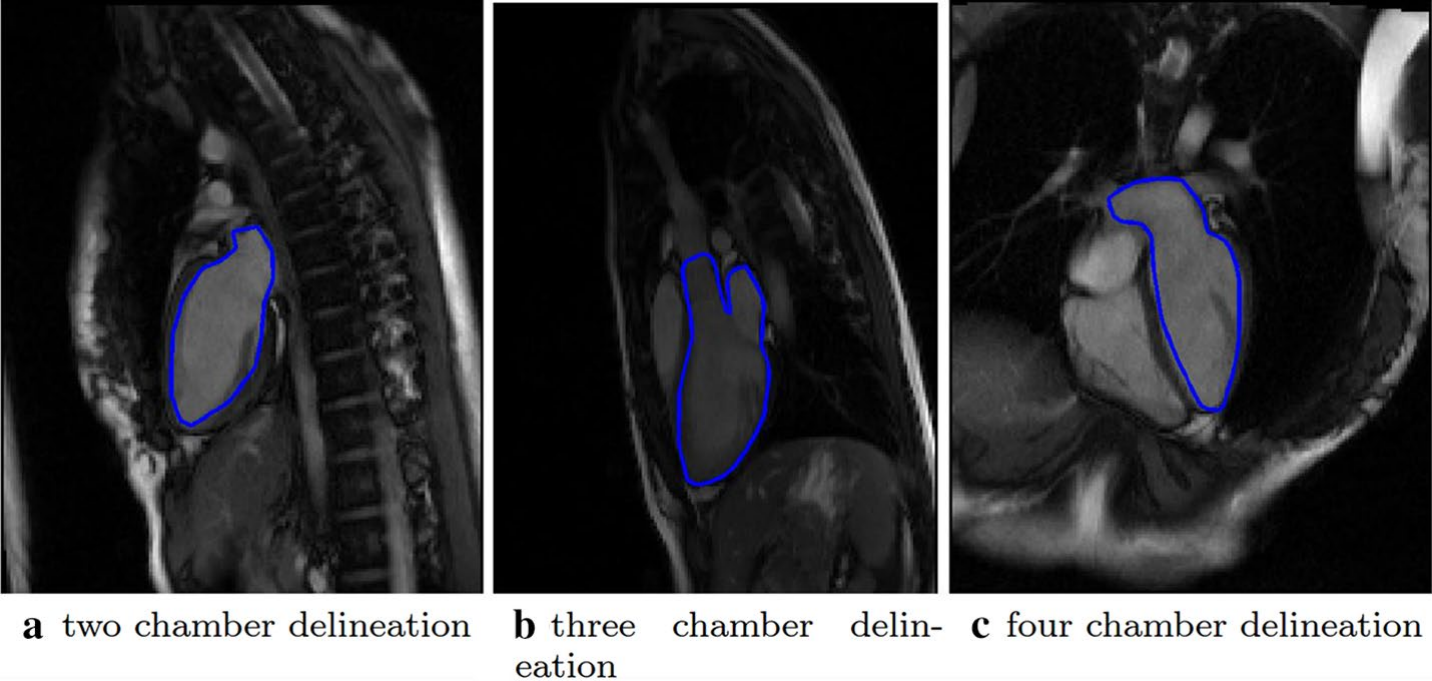
Long axis images and delineated contours. Long axis images in three directions were shown as well as the delineated contours

Typical cine CMR images in the routine clinical imaging protocol include a stack of parallel short-axis image sequences (Fig. 2a–c) ranging from left atrium (LA) and aorta (AO) to the apex of LV and three long axis image sequences, i.e., the two chamber view (Fig. 3a), three chamber view (Fig. 3b), and four chamber view (Fig. 3c). Both long-axis and short-axis images were processed in the *CMRtools suite* (Cardiovascular Solution, UK). Endocardium was delineated by experts for the end-diastole (ED) of each image slice.

For short-axis images, LV or LV inflow and outflow tracts-the left atrio-ventricular junction (AVJ) and the aortic-ventricular junction, or both left atrium (LA) and aorta (AO)-were delineated accordingly (Fig. 2a–c). For the two chamber view, the LV and LA were delineated; for the three chamber view, the LV, LA, and AO were delineated; and for the four chamber view, the LV and LA were delineated (Fig. 3a–c). All papillary muscles were excluded from the myocardial region and partitioned as the blood pool, instead.

The conventional LV modeling method only considers a truncated LV model from the basal level to the apex level. One reason for the incomplete modelling is the difficulty of tackling the inflow/outflow tract and the bifurcation topology. We addressed this issue in our previous study [29] using a variational approach. Delineation not only of the LV but also the LA and AO is a prerequisite for reconstruction of a complete LV model-one includes LV inflow and outflow tracts. A complete LV model provides greater capacity in the quantitative analysis of cardiac function as well as greater credibility in model validation.

The two-dimensional contours delineated from all images were mapped onto three-dimensional space—a patient-based coordinate system-using three imaging specifications: pixel spacing, image position, and image orientation. These image specifications are contained in the DICOM file meta information. The transformation from 2D planar contours to 3D point clouds is as follows.

1 $$\begin{aligned} \begin{bmatrix} x \\ y \\ z \\ 1 \\ \end{bmatrix} = \begin{bmatrix} U_x \triangle u&\quad V_x \triangle v&\quad 0&\quad P_x \\ U_y \triangle u&\quad V_y \triangle v&\quad 0&\quad P_y \\ U_z \triangle u&\quad V_z \triangle v&\quad 0&\quad P_z \\ 0&\quad 0&\quad 0&\quad 1 \\ \end{bmatrix} \begin{bmatrix} u \\ v \\ 0 \\ 1 \\ \end{bmatrix}\,, \end{aligned}$$

where (*u*, *v*) is the 2D coordinate, (*x*, *y*, *z*) is the transformed 3D coordinate, $(P_x,P_y,P_z)$ is the image position (cf. DICOM attribute (0020,0032)), $(U_{x,y,z},V_{x,y,z})$ is the image orientation (cf. DICOM attribute (0020,0037)), and $(\triangle u,\triangle v)$ is the pixel spacing (cf. DICOM attribute (0028,0030)).

**Fig. 4:**
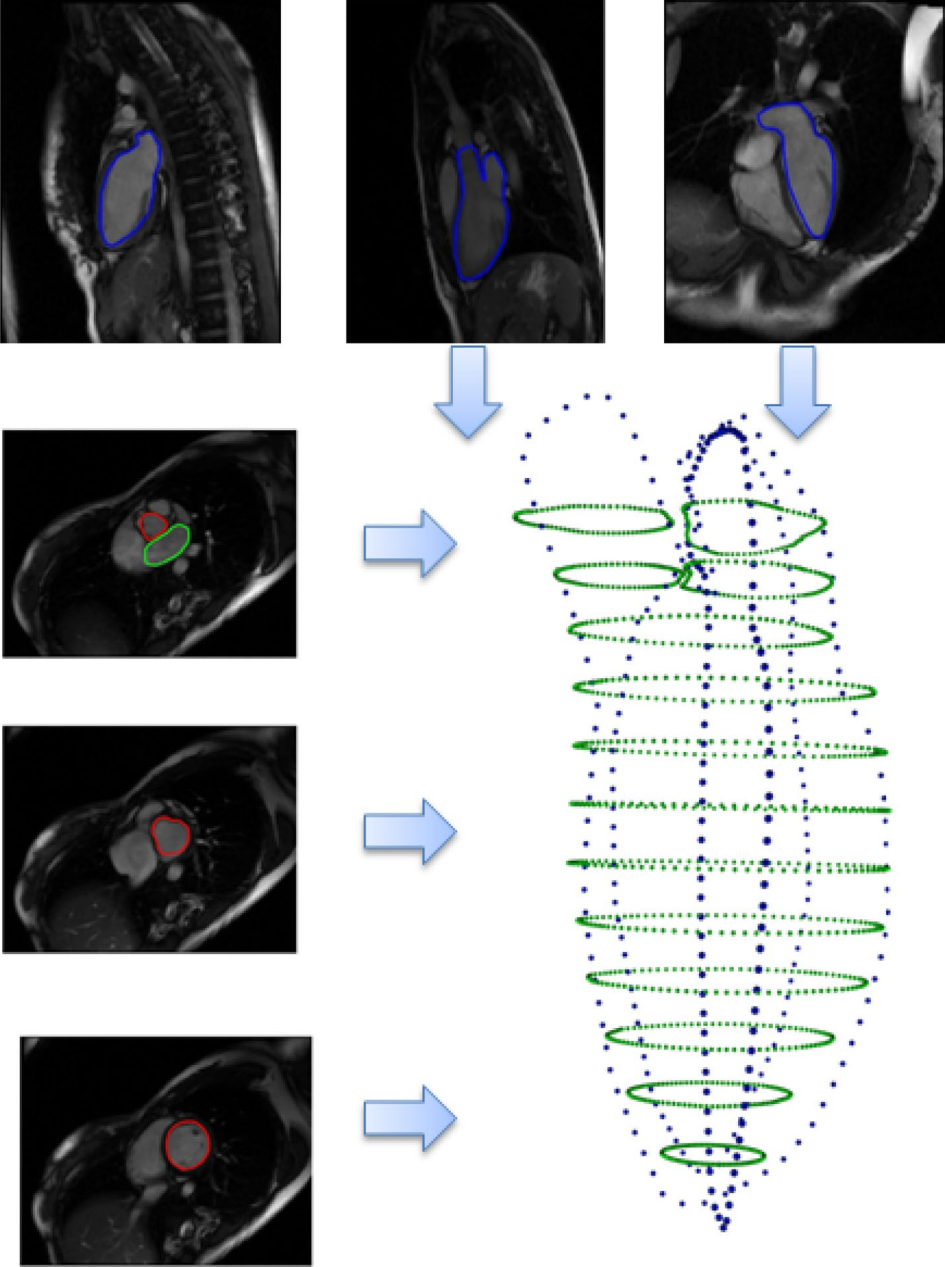
Point cloud from all contours. Contour points from all short and long axis images were projected into the patient-based coordinate system, in which the iterative registration is performed

For computational convenience, all contours were transformed into a position such that the LV is standing on its apex with LA above LV, i.e., valentine position. Figure 4 illustrates points from contours of all images constituting the point cloud, which approximately profiles the entire left heart structure. We denote all contours as follows.

- $C_{sax} = \{C_i, i = 1,\ldots ,L \}$: short-axis contours on *L* parallel short axis slices.
- $C_{lax} = \{C_{2ch},C_{3ch},C_{4ch} \}$: long-axis contours in two-chamber, three-chamber, four-chamber view images.

### Iterative two-step registration

The iterative closest point (ICP) algorithm proposed by Besl and McKay [30] and its variations are widely used to register two sets of points. In this study, we use the generalized ICP [31] for registering a pair of point clouds. The classic ICP estimates the transformation matrix by minimizing the squared distance between these corresponding pairs, while the generalized ICP minimizes the negative log-likelihood of the distance under the assumption that both point clouds are random samples from normally distributed point clouds. We used the generalized ICP based on considerations of possible inaccuracies in contour delineation and presence of high intra- and/or inter-observer variability. The probability-based approach to registering, which assumes sampled points (the manually delineated contours) are drawn from Gaussian distributions centered at the ground truth points (the cavity boundaries), can be expected to largely eliminate inaccuracies introduced at the contour delineation step.

Both the classic and the generalized ICP register a pair of point cloud, i.e., two point clouds. For registration of $C_{sax}$ and $C_{lax}$ involving $L+3$ point clouds, the registering step was divided into two sub-steps:

1. Each contour from a single short-axis slice, $C_i$, was registered against the union of all long-axis contours, $C_{lax}$, and
2. Each contour from a single long-axis slice, $C_{j-ch}$, was registered against the union of all short-axis contours, $C_{sax}$.

**Fig. 5:**
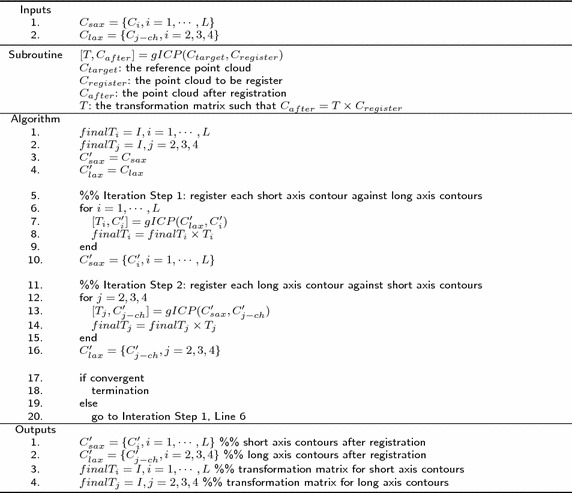
Registration algorithm

These two sub-steps were repeated iteratively until convergence was achieved. This algorithm is described in Fig. 5.

**Fig. 6:**
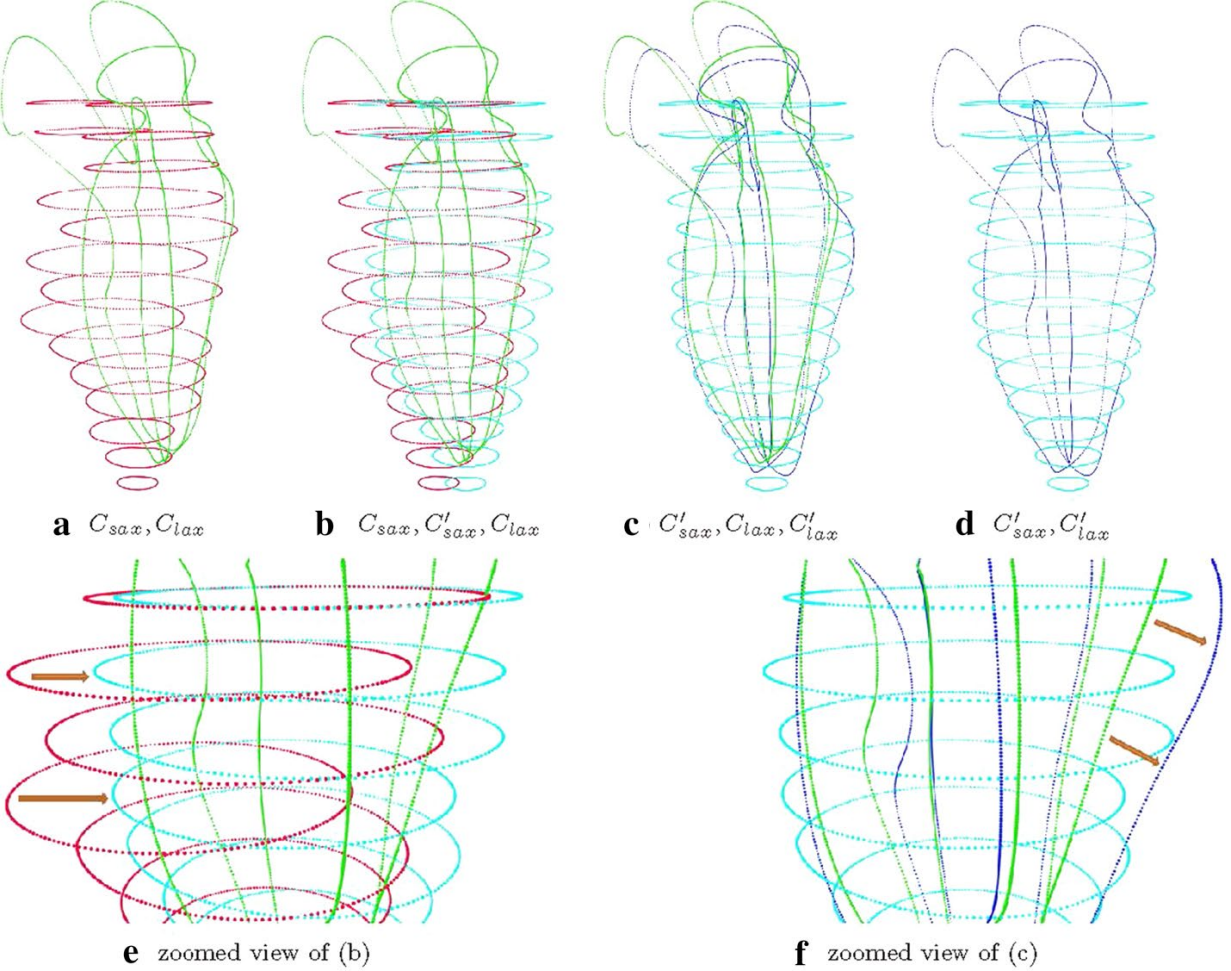
Performing the algorithm in Fig. 5. **a** inputs; **b** intermediate result, at Fig. 5 line 10; **c** intermediate result, at Fig. 5 line 16; **d** outputs; the input contour points (**a**) were iteratively registered to obtain the output contour points (**d**). Two intermediate results were obtained in **b** and **c**. **e** and **f** are zoomed view of two intermedicate results obtained from **b** and **c**, respectively

The transformation used in the registering step is a rigid transformation involving rotation, translation, and composition in three-dimensional space, which comprehends correction of both out-of-plane and in-plane motion distortions. Figure 6 illustrates the steps involved in the registration procedure.

### LV shape reconstruction

As mentioned at the beginning of this section, reconstructing the LV shape provides the basis for validating our motion correction method. Our previous study has already addressed the LV shape reconstruction problem [32]. In this subsection, a brief description of each step is presented, as well as some illustrative information.

The registered point clouds were used to reconstruct the endocardial surface of the left heart. The reconstruction task comprised three steps: (a) interpolation between parallel contour points, (b) tetrahedral mesh generation, and (c) variational mesh segmentation and surface extraction.

**Fig. 7:**
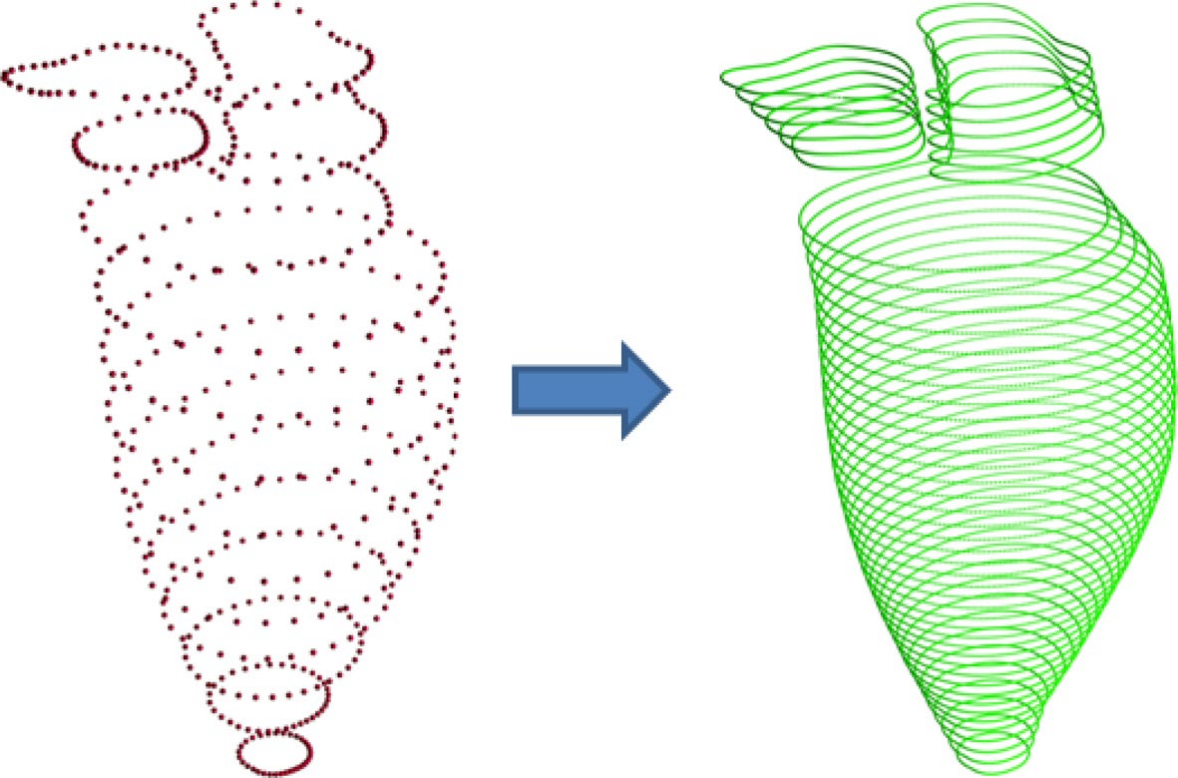
Interpolated point cloud. The registered contour points were interpolated intra-/inter-contours

Interpolation between parallel short-axis contours was carried out which included intra- and inter-contour interpolation (Fig. 7).

**Fig. 8:**
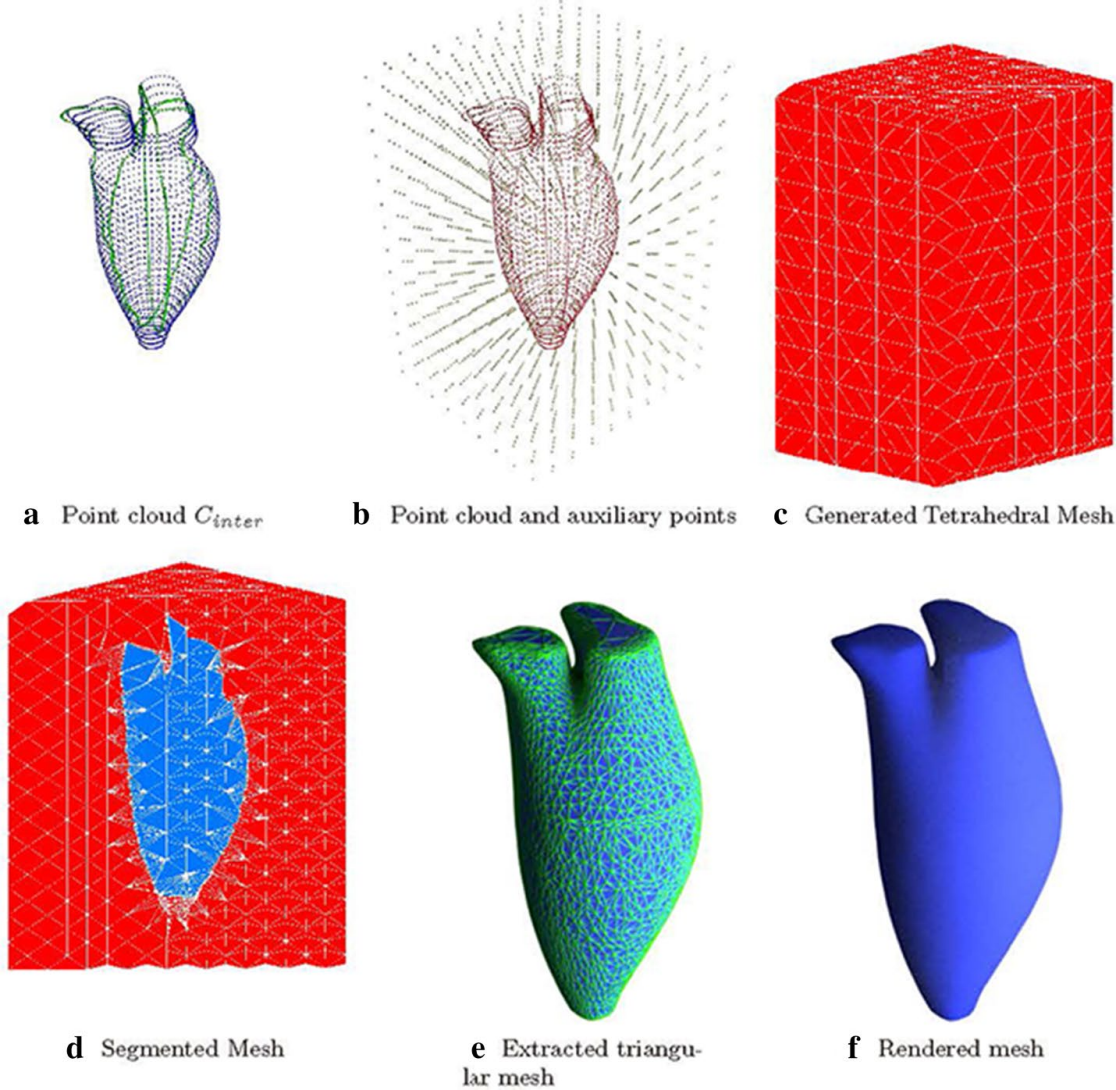
LV shape reconstruction. The interpolated point cloud was utilized to reconstruct the LV shape

The point cloud, $C_{inter}$, (Fig. 8a) was used to generate a Delaunay-based tetrahedral mesh underlying the region of interest. Auxiliary grid points were inserted during the mesh generation procedure. Figure 8b illustrates the preparation for mesh generation: the point cloud, $C_{inter}$, is annotated in red, while the auxiliary point is shown in bright yellow. The selection of auxiliary grid points is described in our previous work [33], where use of a Delaunay-based mesh was also justified (Fig. 8c).

Reconstruction of a triangular mesh surface from the tetrahedral mesh is equivalent to segmenting the tetrahedral mesh into two partitions, interior and exterior. Such a task can be addressed as a variational problem in weighted minimal surface energy [34], i.e.,

2 $$\begin{aligned} E(S) = \int _\Omega d(x,C_{inter}) dx, \end{aligned}$$

where $d(x,C_{inter}) = \min _{y\in C_{inter}} d(x,y)$, *d*(*x*, *y*) is the Euclidean distance between *x* and *y*. The surface *S* minimizing this energy functional is the reconstructed surface.

After discretizing the energy functional (2) on the underlying mesh space, it was noted that the minimization problem could be solved by the graph-cuts technique [35], i.e., a max-flow/min-cut algorithm (Fig. 8d). Applying the graph-cuts technique to the problem, a min-cut was obtained efficiently. A triangular surface mesh was then extracted from the tetrahedral mesh based on the min-cut. After some post-processing—smoothing [36] and re-meshing [37]—a processed left cardiac surface was obtained.

The LV shape reconstruction method was applied to both the un-realigned point cloud and the realigned point cloud with the same parameters. In addition to the visual comparison, quantitative validation is given in "Results" section.

## Results

**Fig. 9:**
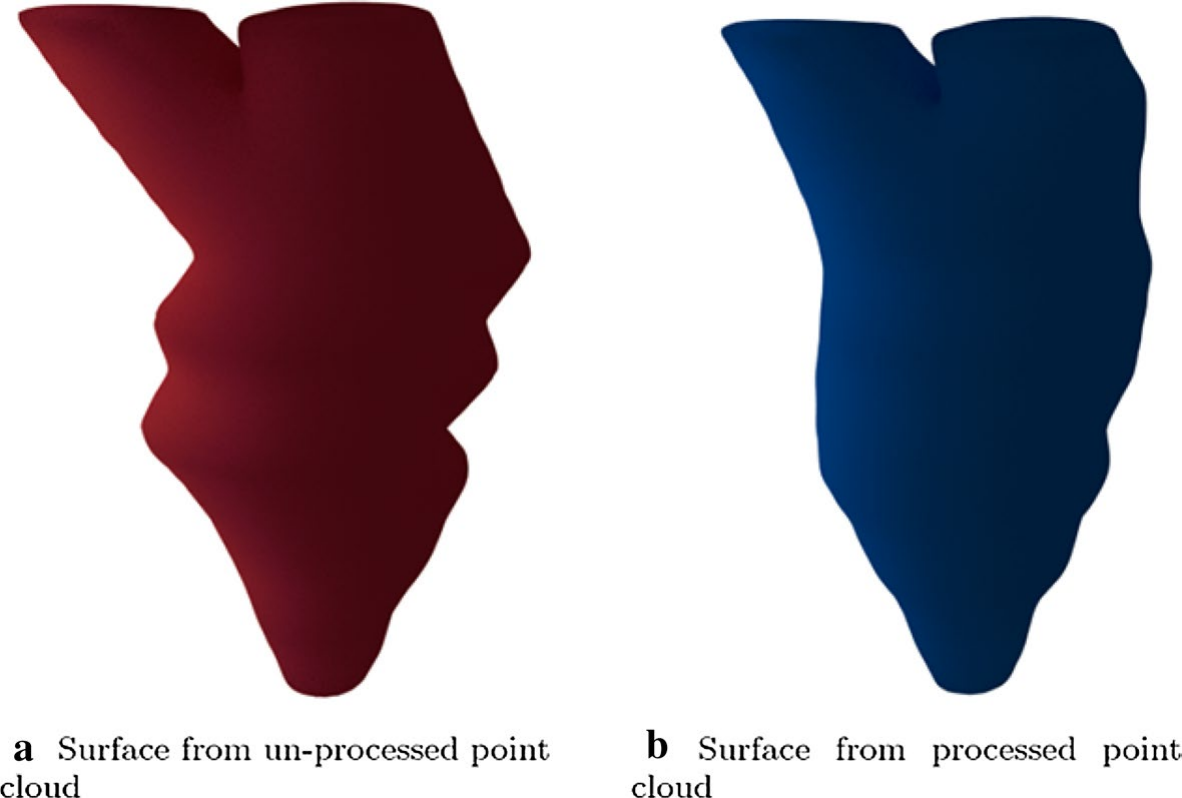
Comparison between un-processed and processed point clouds. A visual comparison between reconstructed models from un-realigned and realigned point clouds

The average time to register the contours in a single case on a 2.5 GHz CPU desktop was about 16 seconds. ED frames were reconstructed for each case. A trained clinician can be expected to delineate a single image in about half a minute. In our study, delineation of a single frame for a single case took about nine minutes. Automatic chamber segmentation (LV and LA segmentation), which could substantially reduce processing time, will be a long-term study issue. The entire processing time (manual delineation, registration, and cardiac modeling) required about ten minutes. Both the triangular mesh and the rendered surface of a single frame are shown in Fig. 8e, f. Reconstruction results are shown in Fig. 9, from which we observe that the unnatural distortion in the un-realigned point cloud is eliminated after image registration realignment.

**Fig. 10:**
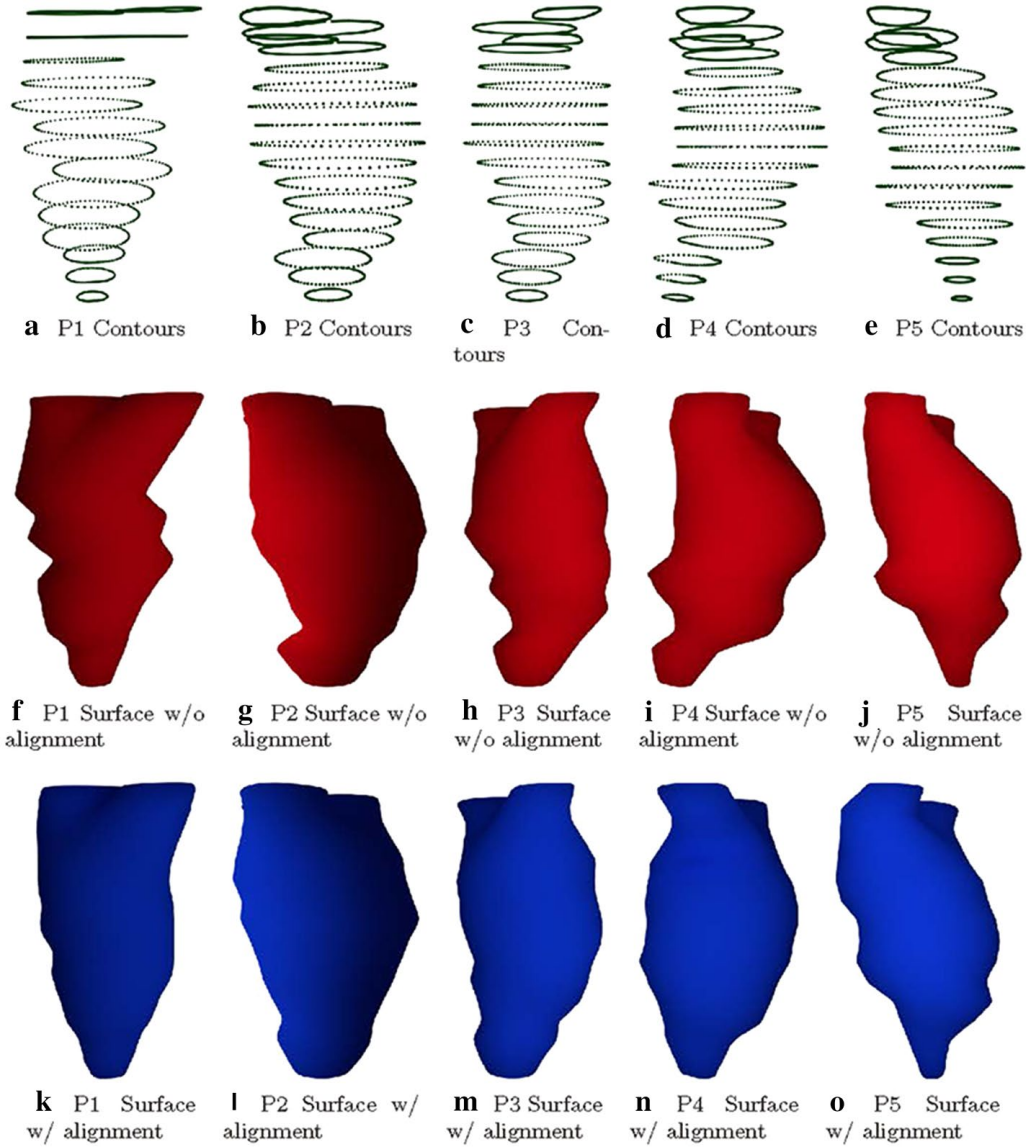
Reconstructed surface model of all subjects. All patients’ un-aligned point clouds were shown in the *first row*; the LV shapes from un-aligned point clouds were shown in the *second row*; the LV shapes from realigned point clouds were shown in the *third row*

For each case, the original contour points, reconstructed surfaces from un-realigned point clouds, and reconstructed surfaces from realigned point clouds are shown in Fig. 10. The top row gives the original contour points, all of which show slice misalignment to a greater of lesser degree. The middle row gives the reconstructed surfaces from un-processed point clouds and highlights the unnatural distortion inherited from the contour points. The bottom row shows the reconstructed surfaces from the processed point clouds. After realignment, distortion in the results has been greatly diminished.

**Fig. 11:**
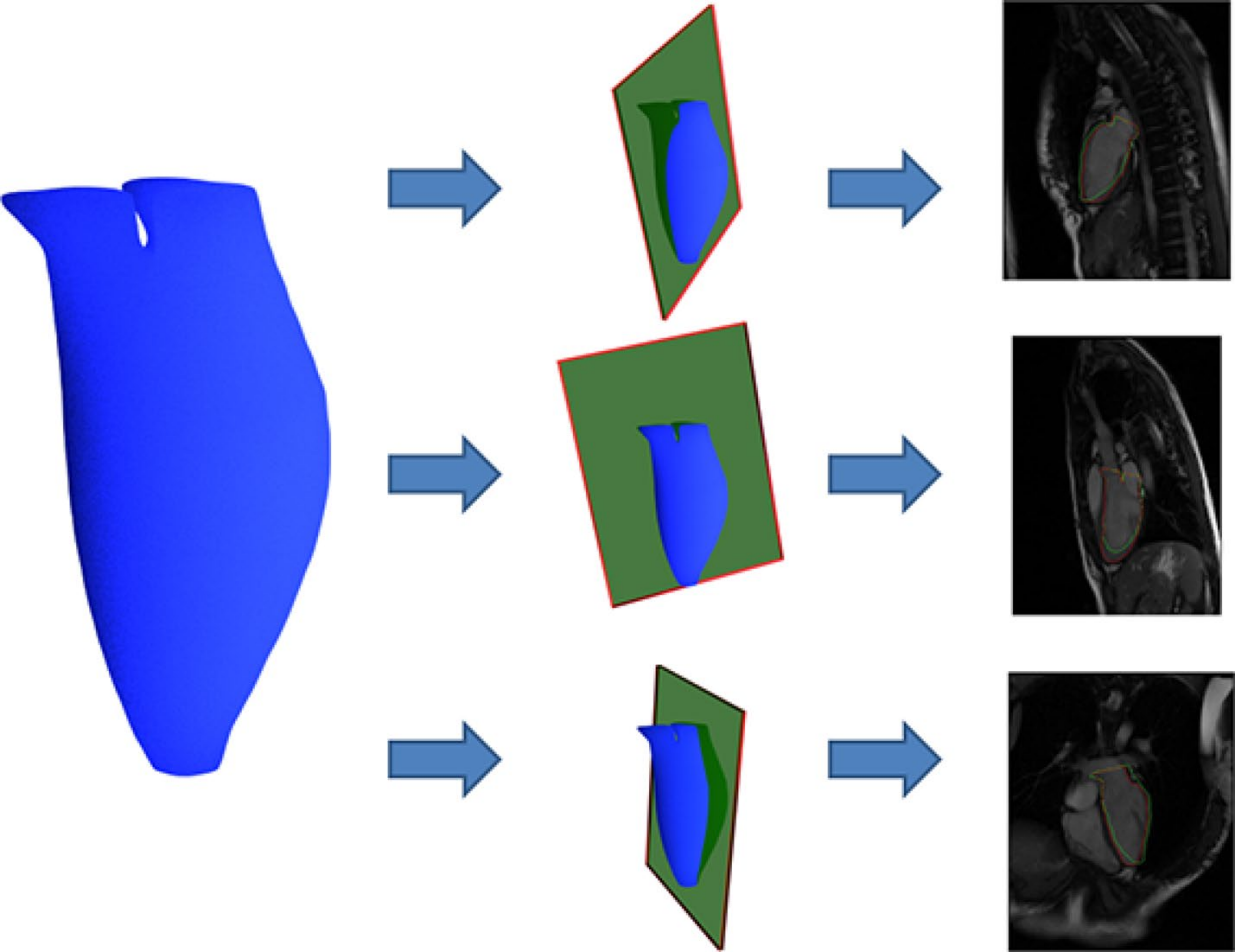
Validation method. Illustration of the validation method: the reconstructed LV shape were intersected with three long axis image planes. The intersection contours were compared with the long axis contours manually delineated

We compared the accuracy of the reconstructed models from point clouds without realignment to those with realignment to assess the effectiveness of our motion correction approach. A gold standard for LV shape in CMR images is not currently available, so the overlap ratio between the reconstructed model and the long-axis contours was used to evaluate the accuracy of the reconstructed shapes. The intersection of the reconstructed surface model and the long-axis imaging planes was computed and validated against contours drawn by experts at the beginning of the study (Fig. 3). This step is illustrated in Fig. 11.

Three criteria were utilized in the assessment: (i) Hausdorff distance, (ii) the Dice similarity coefficient, and (iii) the Jaccard similarity coefficient. Hausdorff distance is a curve-based coefficient that gives the greatest displacement from the reconstructed model to the ground truth contour,

3 $$\begin{aligned} d_H(X,Y) = \inf \{\epsilon \ge 0\,;\ X \subseteq Y_\epsilon \ \text{ and }\ Y \subseteq X_\epsilon \} \end{aligned}$$

**Fig. 12:**
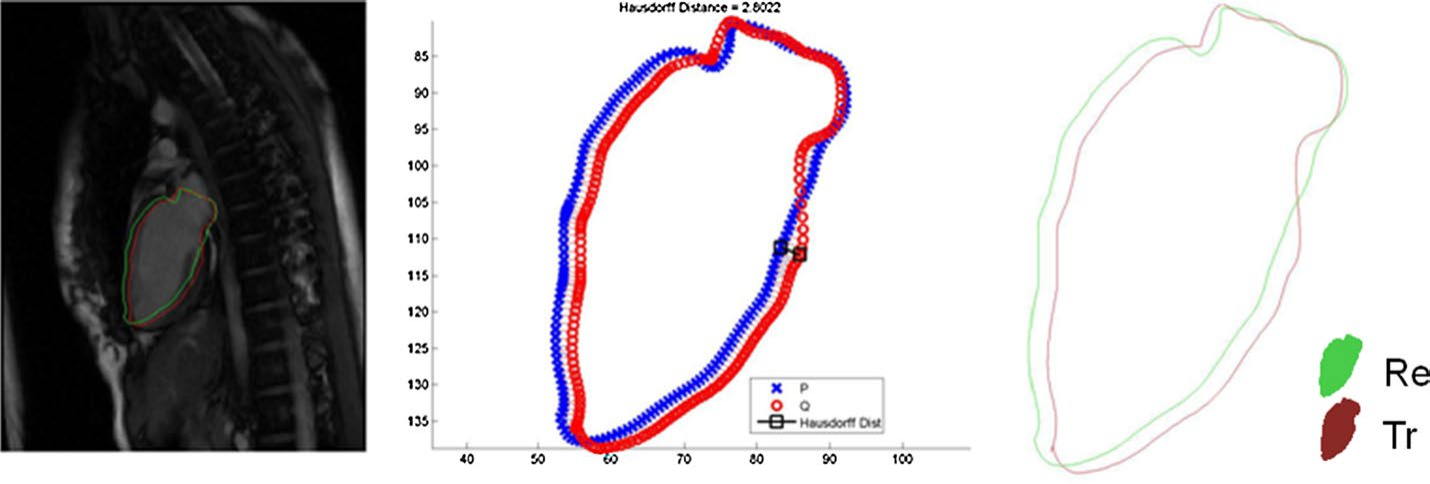
Validation method. *Left* Reconstruction result in *green* and ground truth contour in *red*; *Middle* Hausdorff distance between result and ground truth; *Right* Annotation for Eqs. 4 and 5. Three criteria to evaluate the overlapping ratio between the intersection and the manual delineated contours

Meanwhile, the Dice and Jaccard similarity coefficients are region-based measurements of the overlapping ratio between the reconstructed model and the ground truth contour. The Dice (D) and Jaccard (J) coefficients are defined as follows.

4 $$\begin{aligned} D = \frac{2 \cdot Area(Re \cap Tr)}{Area(Re) + Area(Tr)}\, \end{aligned}$$

5 $$\begin{aligned} J = \frac{Area(Re \cap Tr)}{Area(Re\cup Tr)}\, \end{aligned}$$

where *Re* and *Tr* are regions bounded by the reconstructed model and the contour delineated by experts, respectively. A value of 0.7 and above is considered an adequate overlap [30]. An example of the validation against the two chamber view long-axis contour is shown in Fig. 12.

Validation statistics on reconstruction model results for all five patients are given in Table 2. Un-realigned and realigned results for each case were compared using the three criteria on three long-axis imaging planes. The average evaluation is given in Table 3. For un-realigned patient 3, the reconstructed model has no intersection with the three chamber image, the Hausdorff distance is infinite and the Dice and Jaccard Indices are zeros. From Tables 2 and 3, it is apparent that the motion correction has improved accuracy in the reconstructed results—especially for the region-based criteria. The overall improvement in accuracy realized from the realigned point cloud indicates the effectiveness of the realignment method.

**Table 2:** Validation results for all patients (w/o and w/ realignment)

Patient	Two chamber view			Three chamber view			Four chamber view		
	H (mm)	D	J	H (mm)	D	J	H (mm)	D	J
1 before	12.74	0.89	0.80	9.52	0.89	0.81	9.29	0.95	0.91
1 after	12.35	0.97	0.94	11.70	0.92	0.85	7.59	0.94	0.89
2 before	9.52	0.95	0.91	17.44	0.92	0.85	11.61	0.85	0.74
2 after	11.09	0.97	0.93	14.20	0.88	0.79	12.79	0.96	0.93
3 before	7.97	0.85	0.74	$\infty$	0.00	0.00	18.52	0.73	0.57
3 after	6.93	0.84	0.72	28.37	0.66	0.49	16.90	0.89	0.80
4 before	9.34	0.87	0.77	20.87	0.93	0.87	13.15	0.87	0.76
4 after	5.16	0.94	0.88	3.86	0.93	0.88	4.12	0.93	0.87
5 before	17.88	0.82	0.70	13.96	0.95	0.91	12.25	0.95	0.91
5 after	5.71	0.98	0.97	9.94	0.97	0.94	7.86	0.98	0.95

*H* Hausdorff distance; *D *Dice similarity coefficients; *J* Jaccard similarity coefficients

**Table 3:** Average validation results for all patients (w/o and w/ realignment)

Patient	Mean		
	Mean H(mm)	Mean D	Mean J
1 before	10.52	0.91	0.84
1 after	10.55	0.94	0.89
2 before	12.86	0.91	0.84
2 after	12.69	0.94	0.88
3 before	$\infty$	0.53	0.44
3 after	17.40	0.80	0.67
4 before	14.45	0.89	0.80
4 after	4.38	0.93	0.88
5 before	14.70	0.91	0.84
5 after	7.84	0.98	0.95

## Conclusions

In this study, we propose a novel method to semi-automatically correct or substantially mitigate the effects of breath-hold related and other motions in the cine CMR images. In our approach, LV contours were delineated on both long- and short-axis image planes. Projected into a patient-based coordinate system, all contours were registered using an iterative two-step registration approach based on the generalized ICP algorithm. Contour points with and without motion correction were used to reconstruct the LV shape. Substantially improved accuracy in LV shape based on contours with motion correction indicates the effectiveness of our method. Future relevant work would include a comprehensive validation study against other imaging resource as in [10, 11] and incorporating this geometry-based approach with some image-based approaches. We envision the use of this method in current clinical practice to improve accuracy in the evaluation of cardiac function.

## Abbreviations

CMR: cardiac magnetic resonance
CVD: cardiovascular disease
LV: left ventricle
LA: left atrium
AO: aorta
DICOM: digital imaging and communications in medicine
ICP: iterative closest point
